# Acupuncture at Zuqiaoyin (GB44) Modulates Regional Brain Activity in Migraine Without Aura: A Resting‐State Functional Magnetic Resonance Imaging Study

**DOI:** 10.1155/prm/2433241

**Published:** 2026-09-11

**Authors:** Rong-jiang Yu, Qiang Lin, Cen Zhong, Yi-Chao Chen

**Affiliations:** ^1^ Department of Radiology, Shanghai General Hospital, No. 85/86 Wujin Road Hongkou District, Shanghai, China; ^2^ Department of Acupuncture and Massage, Shanghai General Hospital, No. 85/86 Wujin Road Hongkou District, Shanghai, China; ^3^ Department of Acupuncture and Moxibustion, Dongzhimen Hospital Beijing University of Chinese Medicine, Hai Yun Cang on the 5th Zip Dongcheng District, Beijing, China, bucm.edu.cn

**Keywords:** acupuncture, amplitude of low-frequency fluctuation, migraine, resting-state fMRI, Zuqiaoyin

## Abstract

**Background:**

Migraine is considered a common neurological disorder. Acupuncture at Zuqiaoyin (GB44) is effective in the treatment of migraine without aura, but its central mechanism is still unclear.

**Objectives:**

The purpose is to compare the therapeutic effect and brain functional activity difference between acupuncture at GB44 and nonacupoints and explore the central affect mechanism of acupuncture in the treatment of migraine without aura.

**Methods:**

Our study is a randomized controlled trial. A total of 20 patients with migraine without aura were included in the study. Twelve patients received acupuncture at GB44 as the acupuncture group and eight patients received acupuncture at nonacupoints as the sham acupuncture group. Both groups received a 10‐time acupuncture intervention for 1 month. Visual analogue scale (VAS) score evaluated the clinical outcomes of acupuncture. Resting‐state functional magnetic resonance imaging (fMRI) and fractional amplitude of low frequency fluctuation (fALFF) analysis were adopted to compare differences of brain functional activity between the two groups.

**Results:**

We found that improvements in headache severity and migraine‐related quality of life in both groups after a 4‐week acupuncture intervention but better clinical improvement were observed in the acupuncture group. A decreased fALFF in the right superior frontal gyrus, right insula, and right precuneus and an increased fALFF in the left postcentral gyrus and left precuneus were observed in the acupuncture group, while a decreased fALFF was observed in the left superior frontal gyrus and left precuneus in the sham acupuncture group. The acupuncture group showed a higher fALFF in the right precentral gyrus and right precuneus than the sham acupuncture group.

**Conclusion:**

Acupuncture at GB44 can significantly relieve the clinical symptoms and improve the quality of life of patients with migraine without aura. More importantly, acupuncture at GB44 can cause changes of brain activity in migraine‐affected key regions, which may be one of the central mechanisms of acupuncture in the treatment of migraine without aura.

**Trial Registration:** Chinese Clinical Trial Registry: ChiCTR2000033995

## 1. Introduction

Migraine is a common disease with high morbidity and disability rate [1], ranking second among central nervous system diseases with high economic burden [2]. Migraine without aura (Mwoa) is usually presented as a unilateral throb headache, with each migraine lasting 4–72 h and often accompanied by symptoms such as nausea, vomiting, photophobia, and photophobia [3]. A few migraine patients may also develop other neurological symptoms, such as vertigo, tinnitus, anxiety, and depression [4]. Migraine not only causes substantial personal suffering and reduces quality of life but also has a significant socioeconomic impact [5, 6]. Based on the above points, migraine has gradually become the focus of international attention and research.

Acupuncture is an important part of traditional Chinese medicine. Modern medicine has proved its effectiveness and safety through various studies [7–9]. Meanwhile, a number of high‐quality clinical trials have confirmed that acupuncture is effective in the treatment of migraine [10–13]. For example, a multicenter randomized clinical trial [11] demonstrated the superior efficacy of acupuncture in patients by comparing changes in migraine days and migraine attacks with manual acupuncture, nonpenetrating sham acupuncture, and usual care. The treatment of migraine by acupuncture at Zuqiaoyin (GB44) originates from the root knot theory of acupuncture. Previous studies [14, 15] of our research group have proved that acupuncture of GB44 was effective in the treatment of migraine, but its central mechanism remains unclear.

The development of neuroimaging technology has promoted the research on the pathogenesis of migraine and the central effects of acupuncture. At present, different dimensions and depths have been studied on the onset of migraine and the changes in brain structure and function involved. The exploration of the pathogenesis of migraine at home and abroad has achieved certain consensus results [16–19]. In this study, we adopted the fractional amplitude of low‐frequency fluctuation (fALFF) analysis method to compare the blood oxygen level–dependent (BOLD) signals in the brains of migraine patients in a resting state. Zhang et al. [20] proposed that changes in the fALFF value could reflect changes in the intensity of various voxel spontaneous synchronization neural activities in the low‐frequency range (0.01–0.08 Hz), which was recognized by many researchers. The fALFF analysis method has excellent characteristics of high sensitivity and specificity, which can effectively inhibit nonspecific signals in the brain cisterna and reduce the interference of physiological noise. At the same time, this method can obtain the changed amplitude of BOLD signals relative to baseline from the aspect of energy metabolism and directly suggest the spontaneous electrical activity of neurons [21]. Therefore, the fALFF value has been used as one of the evaluation indices of local spontaneous nerve activity in the resting state [22] and widely applied by clinical researchers in the study of the central mechanism of acupuncture migraine [23, 24].

We hypothesized that acupuncture with GB44 was effective in treating migraine without aura and could affect the functional activity of brain regions to some extent. In this study, we adopted the method of clinical efficacy evaluation and imaging evaluation to observe the neuroimaging alterations induced by acupuncture at GB44 in the treatment of migraine and explore the central affect mechanism of acupuncture in the treatment of migraine.

## 2. Materials and Methods

### 2.1. Ethics Statement

The study will be conducted in accordance with the principles of the Declaration of Helsinki, the Quality Management Practices for Clinical Trials of Medical Devices, and the International Code of Ethics for Research on Human Health. It has been reviewed and approved by the Research Ethical Committee of Dongzhimen Hospital, Beijing University of Chinese Medicine (Approval number: 2022DZMEC‐027‐02). The researchers will follow GCP principles and the approved protocol to implement clinical research and protect the health and rights of each patient. Consent to participate in the study will be obtained using a written informed consent (signature or fingerprint) from all participants recruited.

### 2.2. Participants

From May 2021 to August 2022, migraine patients were recruited from outpatient clinics in the Department of Acupuncture and Moxibustion of Dongzhimen Hospital, Beijing University of Chinese Medicine, by displaying recruitment posters outside the clinics. All participants gave written informed consent prior to inclusion.

According to the diagnostic criteria for migraine without aura in the International Classification of Headache Disorders, Third Edition (ICHD‐3) [3], published by the International Headache Society in 2018, Migraine without aura is diagnosed with at least five migraine attacks that meet the following three criteria: (1) Headache attacks lasting 4–72 h (untreated); (2) headache has at least two of the following four characteristics: a. unilateral; b. pulsation; c. moderate or severe headache; d. daily activities (such as walking or climbing stairs) can aggravate headache, or actively avoid such activities when headache; and (4) at least one of the following symptoms occurs during headache: (a) nausea and/or vomiting; (b) photophobia and photophobia.

Referring to the diagnostic criteria and the purpose of this research, we have formulated the following strict inclusion and exclusion criteria. The inclusion criteria for migraine patients were as follows: (1) The patient was diagnosed with migraine without aura based on ICHD‐3, and the headache was located on the side of the head; (2) age 18–65 years old, right‐handed; (3) a history of migraine for at least 1 year; (4) migraine attacks at least twice a month; (5) no acupuncture or drug treatment was used within 24 h after the acute onset of migraine, and no preventive treatment for migraine in the past 3 months; and (6) the subject signed the informed consent and volunteered to participate in the experiment.

The criteria for exclusion were as follows: (1) Tension headache, neurotic headache, cluster headache, and other primary headache; (2) suffering from other secondary headaches or other neurological diseases, such as ear, nose, and throat diseases or headaches caused by depression, Parkinson’s disease, and other intracranial lesions; (3) more serious systemic diseases (cardiovascular diseases, acute infectious diseases, blood diseases, endocrine diseases, allergies, or hemoptysis); (4) patients who have used preventive drugs or acupuncture treatment in the last 3 months; (5) pregnancy, lactation, and pregnancy preparation; (6) smoking, alcohol, and drug users; (7) patients with pain within 3 days before the scan; (8) people with acupuncture contraindications, such as blood diseases, local infections, etc.; and (9) people with nuclear magnetic scanning contraindications, such as metal implantation, claustrophobia, etc.

### 2.3. Study Design

We performed a single‐blind, randomized controlled trial with two groups: acupuncture group and sham acupuncture group. The purpose of our research is to explore the central effect mechanism of acupuncture at Zuqiaoyin (GB44) in the treatment of migraine without aura on the basis of a comprehensive evaluation of pain degree, quality of life, acupuncture reliability, and validity in patients with migraine without aura, combined with resting state functional magnetic resonance technology.

Before participating in the study, all patients were required to fill in a series of questionnaires to know their age, education level, and headache course and recall the severity of pian and quality of life over the past 4 weeks. Eligible patients were randomly assigned to the acupuncture group or sham acupuncture group for acupuncture intervention of 4 weeks. Meanwhile, the changes of brain function and clinical symptoms were compared and analyzed by fMRI scanning and scale evaluation before and after acupuncture intervention.

### 2.4. Randomization and Blinding

In this study, eligible migraine patients without aura were randomly divided into two groups according to the random number table method. The specific methods were as follows: SAS statistical software (SAS Version 9.0, SAS Institute, Inc., Cary, NC) was used to generate random number table, and sealed randomized envelope was made (the light‐tight random envelope was designed in advance, the serial number was affixed to the outside of the envelope, and the random number and group were sealed inside). Patients were assigned to the acupuncture group and sham acupuncture group based on the generated random number table.

This study was a single blind trial in which patients were blinded. To keep migraine patients blind during treatment, migraine patients in both groups were told that they would receive one of two acupuncture treatments, which depended on different traditional Chinese acupuncture theories. In addition, acupuncture intervention was performed in a consulting room, separated by screens to protect patients’ privacy and patients of both groups received the same amount of bilateral acupoint stimulation each time.

### 2.5. Intervention

In this study, acupuncture intervention was manipulated by two specialized acupuncturists. Both of the acupuncturists had more than 2 years of experience in acupuncture and had received systematic acupuncture training before conducting the study to ensure the consistency of acupuncture manipulation and acupoint positioning. They would take turns performing acupuncture interventions for patients.

**FIGURE 1:**
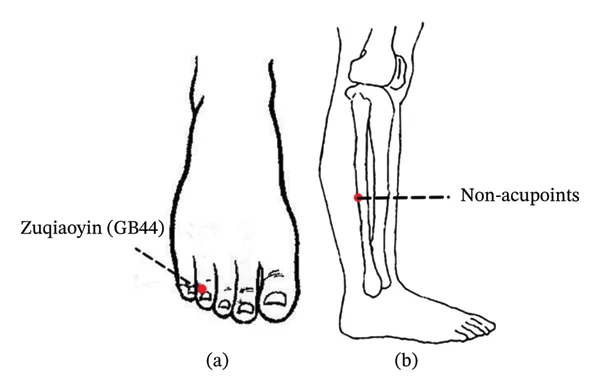
(a) Location of Zuqiaoyin (GB44) in the acupuncture group; (b) location of nonacupoints in the midpoint of the line between Fenglong (ST40) and Chengshan (BL57) in the sham acupuncture group.

Acupoint selection of the acupuncture group will be based on the “root knot” theory of traditional Chinese medicine, and Zuqiaoyin (GB44) was selected as the acupuncture point of the acupuncture group (see Figure 1). Prior to this study, the effectiveness of GB44 in the treatment of migraine has been proved by several studies [14, 15]. In the sham acupuncture group, the midpoint of the line between Fenglong (ST40) and Chengshan (BL57) was used as nonacupoints (see Figure 1). The location of acupoints refers to the national standard “Name and Location of Meridian Acupoints,” standard number: GB/T12346‐2021.

To simulate real‐world acupuncture treatment, the total acupuncture intervention of two groups was continuous 4 weeks for each migraine patient, including three times a week for the first 2 weeks and two times a week for the second 2 weeks. After routine disinfection of acupoints, all acupoints were punctured bilaterally using disposable sterile acupuncture needles (Hwato Needles, Sino‐foreign Joint Venture Suzhou Hua Tuo Medical Instruments Co., China), 25 mm in length and 0.25 mm in diameter. The depths of the inserted needles were approximately 0.2 cm–0.5 cm. The needle is inserted 90° perpendicular to the skin surface. After acquiring the de‐qi sensation, acupuncturists would perform even reinforcing‐reducing manipulation for 5 s and each acupuncture intervention lasted for 20 min (“de‐qi sensation” is a reaction of acid, swelling, numbness, and heaviness at the acupuncture site of the patient. Even reinforcing‐reducing manipulation is to implement even and peaceful needle movement after obtaining de‐qi sensation from acupuncture). All patients were in supine or seated position during acupuncture. It is worth mentioning that ibuprofen can be used for analgesic treatment when acute migraine attacks occur and the specific situation needs to be recorded in a headache diary in detail.

### 2.6. Outcome Measures

#### 2.6.1. Clinical Efficacy Evaluation

All patients filled in headache diaries during the trial. The purpose of a headache journal is to record detailed information about migraine attacks, including their frequency, the severity of the pain, and the use of acute pain medications (it was prescribed that 300 mg of ibuprofen sustained release capsules can be taken when headache attacks are unbearable).

A visual analogue scale (VAS) score of 0–10 was considered a primary clinical outcome to assess the severity of pain. Patients were asked to recall their headache in the past 4 weeks before enrollment and fill in the VAS score according to the severity of headache. Researchers calculated the mean used as the baseline average VAS score. Similarly, the VAS scores of each headache attack during acupuncture were also collected and calculated as the average VAS score after a 4‐week acupuncture.

Secondary measures to evaluate the efficacy of acupuncture included headache diary data, such as changes in the frequency and days of migraine attacks before and after acupuncture. In addition, the Migraine specific Quality of Life Questionnaire (MSQ) and Headache Impact Test‐6 (HIT‐6) were used to evaluate the changes in patients′ quality of life before and after acupuncture.

#### 2.6.2. Evaluation of Acupuncture Reliability and Validity

It is worth mentioning that acupuncture credibility score and expectancy evaluation were recorded for each patient before treatment to know each patient’s expectation for acupuncture treatment of migraine and their degree of trust in acupuncture treatment.

#### 2.6.3. Safety Evaluation

After each treatment, acupuncture‐related adverse events were recorded, including acupuncture dizziness, acupuncture stagnation, unbearable acupuncture pain (VAS ≥ 8), local hematoma, infection, and other discomfort symptoms. Researchers should record adverse events in detail including the severity and outcome of adverse events and measures taken for adverse reactions.

### 2.7. Magnetic Resonance Imaging (MRI) Data Acquisition

All MRI scanning was conducted on a Siemens 3.0 T scanner (Skyra, Siemens, Erlangen, Germany) in the Department of Radiology for the Beijing Hospital of Traditional Chinese Medicine Affiliated to Capital Medical University for acquiring better MRI data. Before MRI scanning, participants were asked to check any carryon magnetic metal items and electronic equipment to keep safety and then placed in a supine position with their head snugly fixed by straps and foam pads to minimize head movement. During scanning, all participates were instructed to stay awake and remain as motionless as possible. When receiving the instruction, participants need to keep an eye on a cross‐shaped icon. Every scanning session lasted approximately 30 min. A high‐resolution T1‐weighted magnetization–prepared rapid gradient echo (MPRAGE) sequence was acquired and covered the entire brain [192 sagittal slices, repetition time (TR) = 2300 ms, echo time (TE) = 2.32 ms, field of view (FOV) = 240 × 240 mm, flip angle = 8°, acquisition matrix = 256 × 256, 290 scans]. Rs‐fMRI data were acquired using a single shot gradient echo‐planar imaging (EPI) sequence, during which 240 EPI volumes were obtained (40 axial slices, TR = 3000 ms, TE = 30 ms, FOV = 220 × 220 mm, flip angle = 90°, matrix = 256 × 256).

### 2.8. Data Analysis

#### 2.8.1. Demographic and Clinical Data Analysis

All statistical analyses were performed using IBM SPSS Statistics version 25.0 (IBM SPSS Inc., Chicago, IL, USA). The statistical significance threshold was set at *p* < 0.05, and all tests were two‐tailed. Normality of data distribution was assessed using the Shapiro–Wilk test in conjunction with histogram inspection. Continuous variables with a normal distribution were compared between groups using the independent samples *t* test, whereas non‐normally distributed data were analyzed using the Mann–Whitney *U* test. Categorical variables, such as sex ratio, were compared between groups using the chi‐squared (*χ* ^2^) test. To compare changes in outcome measures across time and between groups, a mixed‐design analysis of variance (ANOVA) was performed, with time (pretreatment, post‐treatment) as the within‐subjects factor and group (acupuncture, sham acupuncture) as the between‐subjects factor; the time × group interaction was reported. Where a significant interaction was found, post hoc within‐group pairwise comparisons were conducted using paired *t* tests; the Wilcoxon signed‐rank test was used when the difference scores were not normally distributed.

#### 2.8.2. Imaging Data Preprocessing

In order to rule out macrostructural brain lesions that may affect the brain microstructure or function, all of the conventional T1‐weighted imaging had been reviewed before preprocessing by two senior radiologists in the Department of Radiology of the Beijing Hospital of Traditional Chinese Medicine Affiliated to Capital Medical University. All of the high‐resolution T1‐weighted images were displayed and carefully checked, and functional images were checked by MRIcro software (https://www.MRIcro.com) to exclude potential low image quality by two authors. No participants were excluded because of brain lesions or low image quality.

The resting‐state functional and structural images were preprocessed using Statistical Parametric Mapping (SPM8) (https://www.fil.ion.ucl.ac.uk/spm). The first 10 functional volumes of each participant were removed to allow for the participants’ adaptation to the scanning noise and the stability of the initial signal and finishing of slice timing. A three‐dimensional head motion correction was conducted for the remaining time points. None of the participants were removed according to the head motion criteria, which included a maximum spin (*x*, *y*, *z*) of less than 2.0° and a maximum cardinal direction displacement (*x*, *y*, *z*) of less than 2.0 mm. The Diffeomorphic Anatomical Registration Through Exponentiated Lie Algebra tool was used to compute transformations from native space to Montreal Neurological Institute (MNI) space and resampled to 3 × 3 × 3 mm voxels. Subsequently, the white matter signal, cerebrospinal fluid signal, global signal, and Friston 24‐parameter were regressed from the time series of all voxels via linear regression.

#### 2.8.3. MRI Data Analysis

Amplitude of low‐frequency fluctuation (ALFF) is an image analysis method that reflects the degree of spontaneous activity of neurons within a certain time. It is generally believed that low‐frequency drift is less than 0.01 Hz and physiological noise is greater than 0.1 Hz. In order to reduce the influence of noise on ALFF, the ratio of ALFF to the fluctuation amplitude of the full frequency range (0–0.25 Hz) is used as an indicator to measure the ALFF, which is called fALFF. In this study, the fALFF calculation was performed using the Data Processing & Analysis for Brain Imaging (DPABI) software (version 6.1, Beijing, China; https://rfmri.org/DPABI) and fALFF value was calculated in the following steps: (1) Frequency domain transform: Fourier transform is performed on the fMRI time series to convert each voxel’s signal from the time domain to the frequency domain; (2) amplitude calculation: the power spectrum of the full‐frequency signal (0–0.25 Hz) is obtained, and the amplitude is derived by taking the square root of the power spectrum (since power is proportional to the square of the amplitude); (3) ALFF computation: the ALFF value is obtained by summing the amplitude at each frequency point within the low‐frequency band (0.01–0.08 Hz) and dividing by the number of time points; (4) fALFF calculation: the fALFF value is derived as the ratio of the ALFF (the summed amplitude within 0.01–0.08 Hz) to the total amplitude summed across the entire frequency range (0–0.25 Hz). [25–27]. As the fALFF denominator required the full‐frequency signal, the fALFF calculation was performed on the unfiltered residual time series, and a temporal band‐pass filter (0.01–0.08 Hz) was subsequently applied to the residual signal to reduce low‐frequency drift and high‐frequency noise before the voxel‐wise statistical analysis.

In the Statistical Analysis module of DPABI, analysis of covariance (ANCOVA) was performed to compare postintervention fALFF values between groups, with preintervention fALFF included as a covariate. Subsequently, paired‐sample *t*‐tests were used to compare fALFF values before and after the intervention within each group. The Gaussian random field (GRF) tool was used for multiple comparison correction. The *p* value of Voxel was 0.05, and the *p* value of Cluster was 0.05. DPABI viewer was used for visualization analysis.

## 3. Results

### 3.1. Demographic and Baseline Clinical Data

**FIGURE 2:**
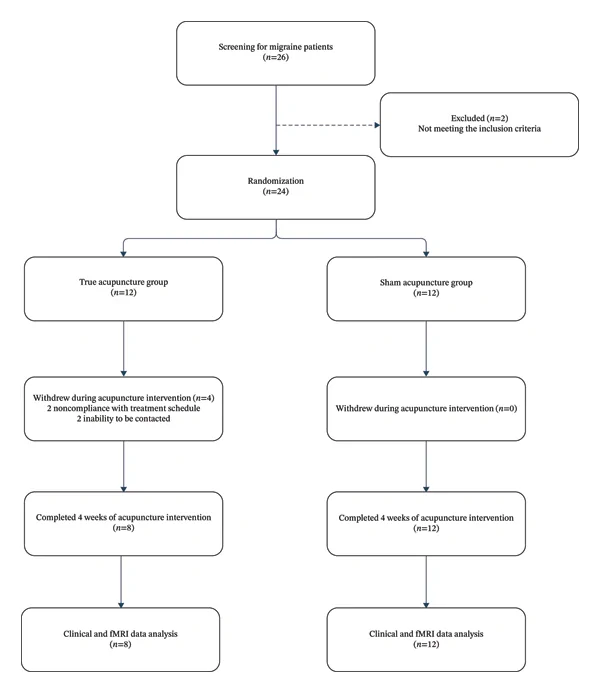
The flow chart of the study.

Twenty‐four eligible patients with migraine without aura were equally allocated into the acupuncture group and sham acupuncture group. Four patients from the sham acupuncture group dropped out during a 4‐week acupuncture intervention because of personal reasons, such as noncompliance with the treatment schedule and inability to be contacted (Figure 2). Therefore, a total of 20 patients with migraine without aura were involved in the analysis, including 12 from the acupuncture group and 8 from the sham acupuncture group. According to the analysis of Edinburgh Handedness Inventory [28], all patients were right‐handed. Demographic and clinical data of two groups are presented in Table 1, which shows that the demographic data and baseline clinical data of the two groups were comparable (*p* > 0.05).

**TABLE 1:** Demographic and clinical traits of all migraine patients without aura.

Items	Acupuncture group (*n* = 12)	Sham acupuncture group (*n* = 8)
Female, *n* (%)	11 (91.67%)	7 (12.50%)
Mean age (SD), (years)	29.17 (6.94)	35.63 (10.72)
Mean weight (SD), (kg)	57.00 (6.95)	62.50 (10.95)
Mean height (SD), (cm)	164.33 (7.06)	165.00 (7.87)
Mean education (SD), (years)	18.17 (2.08)	16.63 (1.69)
Mean duration of illness (SD), (years)	8.08 (4.79)	10.38 (10.17)
Acupuncture reliability score (SD)	21.58 (4.01)	22.38 (1.92)
Expectancy evaluation (SD)	0.70 (0.15)	0.70 (0.11)

*Note:* Data are presented as mean (SD).

Abbreviation: SD, standard deviation.

### 3.2. Changes of Clinical Outcome in Two Groups After 4‐Week Acupuncture

As shown in Table 2, the number of days, frequency, and VAS score of migraine were significantly decreased in the acupuncture group after 4 weeks of acupuncture (*p* < 0.05). Significant changes in HIT‐6 and MSQ scores indicated that the quality of life was improved after a 4‐week acupuncture on GB44 (*p* < 0.05). Different from the acupuncture group, only significant changes in the VAS score were observed in the sham acupuncture group after 4 weeks and no significant changes were shown in migraine days and frequency. In terms of quality of life, patients in the sham acupuncture group showed significant changes in HIT‐6 and MSQ‐R scores, but no significant changes in other parts.

**TABLE 2:** Clinical outcome measures compared between the acupuncture and sham acupuncture groups.

	Acupuncture group (*n* = 12)				Sham acupuncture group (*n* = 8)				Mixed‐design ANOVA (group × time interaction)	
	Before acupuncture (SD)	After acupuncture (SD)	Test statistics	*p* ^∗^	Before acupuncture (SD)	After acupuncture (SD)	Test statistics	*p* ^∗^	*η* ^2^ *p*	*p* ^∗∗^
Number of days with migraine (days)	4.67 (2.23)	2.50 (0.80)	*t* = 3.532	0.005	2.88 (0.84)	3.00 (2.78)	*t* = ‐0.146	0.888	0.218	0.038
Frequency of migraine attacks	3.83 (1.75)	2.42 (0.79)	*t* = 3.261	0.008	3.13 (0.84)	2.25 (1.91)	*t* = 1.178	0.277	0.686	0.001
Average VAS score	6.75 (1.31)	4.03 (1.73)	*t* = 6.396	0.000	6.38 (1.77)	3.41 (2.55)	*t* = 3.234	0.014	0.311	0.011
HIT‐6	64.33 (5.57)	53.25 (7.24)	*t* = 5.054	0.000	65.38 (5.85)	53.75 (7.29)	*Z* = −2.384	0.017	0.737	0.000
MSQ‐E	64.81 (8.97)	73.61 (10.63)	*t* = −2.994	0.012	65.97 (14.07)	69.44 (14.85)	*Z* = 1.198	0.231	0.292	0.014
MSQ‐P	50.00 (10.81)	64.58 (15.23)	*t* = −3.437	0.015	44.79 (14.22)	59.37 (14.56)	*t* = −2.246	0.060	0.415	0.002
MSQ‐R	51.39 (10.23)	64.88 (12.48)	*t* = −2.957	0.013	47.02 (12.58)	58.04 (14.23)	*t* = −2.885	0.023	0.447	0.001

*Note:* Data are presented as mean (SD). HIT‐6, Headache Impact Test Questionnaire‐6; MSQ‐E, Migraine‐Specific Quality‐of‐Life Questionnaire‐Emotional Function; MSQ‐P, Migraine‐Specific Quality‐of‐Life Questionnaire‐Role Function‐Preventive; MSQ‐R, Migraine‐Specific Quality‐of‐Life Questionnaire‐Role Function‐Restrictive.

Abbreviations: SD, standard deviation; VAS, visual analogue scale.

^∗^ *p* values for within‐group comparisons between pre‐ and post‐treatments, derived from paired *t*‐tests (or the Wilcoxon signed‐rank test when difference scores were not normally distributed).

^∗∗^ *p* values for the group × time interaction, derived from the mixed‐design ANOVA.

The mixed‐design ANOVA revealed significant group × time interactions for the number of days with migraine (*η* ^2^ *p* = 0.218, *p* = 0.038), the frequency of migraine attacks (*η* ^2^ *p* = 0.686, *p* = 0.001), the average VAS score (*η* ^2^ *p* = 0.311, *p* = 0.011), the HIT‐6 (*η* ^2^ *p*=0.737, *p* < 0.001), the MSQ‐E (*η* ^2^ *p*=0.292, *p* = 0.014), the MSQ‐P (*η* ^2^ *p*=0.415, *p* = 0.002), and the MSQ‐R (*η* ^2^ *p*=0.447, *p* = 0.001). These results indicate that the changes in clinical outcome measures over time differed significantly between the acupuncture and sham acupuncture groups.

### 3.3. Changes of fALFF in Two Groups

**FIGURE 3:**
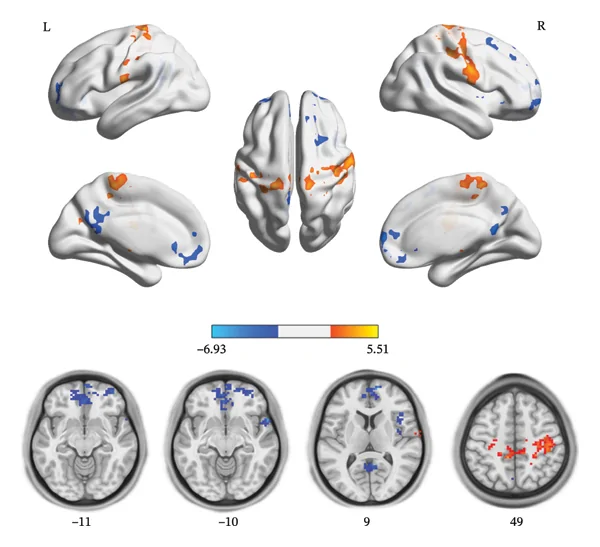
fALFF changes in the true acupuncture group after a 4‐week acupuncture. The hot (cool) color indicates a significantly increased (decreased) fALFF brain area.

In the acupuncture group, migraine patients showed decreased fALFF value in the right superior frontal gyrus, right insula, and right precuneus after a 4‐week acupuncture intervention. An increased fALFF was observed in the left postcentral gyrus and left precuneus compared with that before acupuncture (Table 3 and Figure 3).

**TABLE 3:** The fALFF changes of migraineurs in the acupuncture group after a 4‐week acupuncture.

Sign	L/R	Brain regions	MNI coordinates			Intensity	Cluster size (voxel)
			*X*	*Y*	*Z*		
↓	R	Superior frontal gyrus	9	60	9	−5.0464	20
↓	R	Insula	42	3	0	−6.6538	46
↓	R	Precuneus	3	−51	15	−5.5993	52
↑	L	Postcentral gyrus	−52	−12	18	4.2591	122
↑	L	Precuneus	0	−36	57	6.5065	17

*Note:* Up or down arrow (↑/↓) indicates whether the fALFF signal increases or decreases, respectively, L (R), left (right) cerebral hemisphere.

**FIGURE 4:**
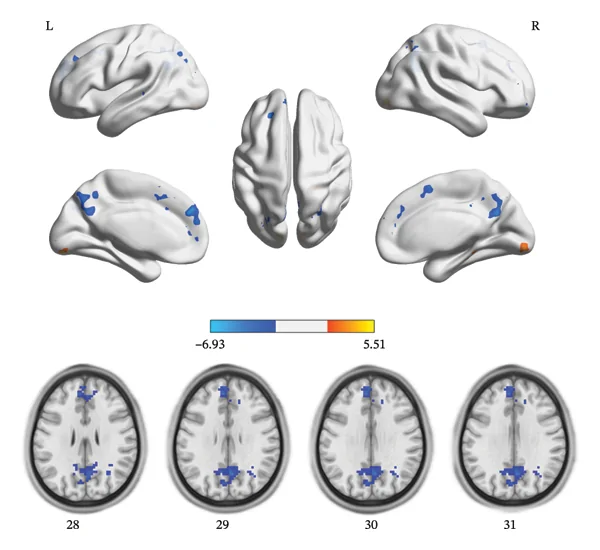
fALFF changes in the sham acupuncture group after a 4‐week acupuncture. The hot (cool) color indicates a significantly increased (decreased) fALFF brain area.

In the sham acupuncture group, the fALFF value showed a decrease in the left superior frontal gyrus and left precuneus. No brain regions showed increased fALFF values (Table 4 and Figure 4).

**TABLE 4:** The fALFF changes of migraineurs in the sham acupuncture group after a 4‐week acupuncture.

Sign	L/R	Brain regions	MNI coordinates			Intensity	Cluster size (voxel)
			*X*	*Y*	*Z*		
↓	L	Superior frontal gyrus	−6	48	33	−6.4467	77
↓	L	Precuneus	−3	−57	51	−9.8264	132

*Note:* Up or down arrow (↑/↓) indicates whether the fALFF signal increases or decreases, respectively, L (R), left (right) cerebral hemisphere.

**FIGURE 5:**
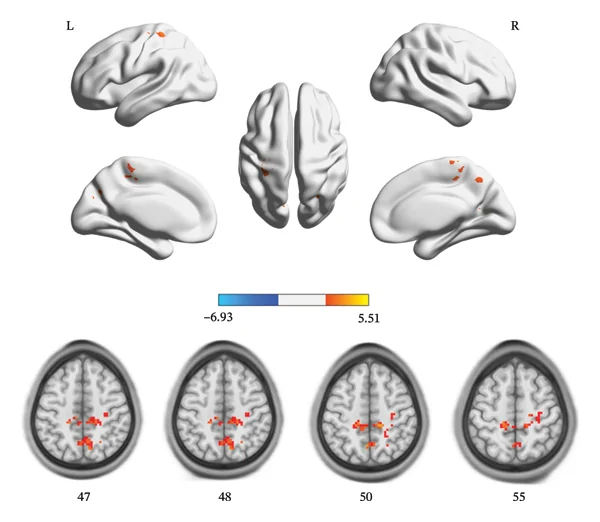
Comparison of fALFF changes between two groups after a 4‐week acupuncture. The hot color indicates a significantly higher fALFF of brain regions in the true acupuncture group.

The comparison of brain areas between the two groups after 4 weeks of acupuncture intervention is as follows: compared with the sham acupuncture group, the acupuncture group showed a higher fALFF value in the right precentral gyrus and right precuneus. Detailed results are shown in Table 5 and Figure 5.

**TABLE 5:** Comparison of fALFF between acupuncture group and sham acupuncture group after a 4‐week acupuncture.

Condition	L/R	Brain regions	MNI coordinates			Intensity	Cluster size (voxel)
			*X*	*Y*	*Z*		
Acupuncture > Sham acupuncture	R	Precentral Gyrus	15	−33	51	4.0562	53
Acupuncture > Sham acupuncture	R	Precuneus	12	−66	45	5.5104	54

*Note:* L (R), left (right) cerebral hemisphere.

### 3.4. Adverse Events

All patients in our study did not suffer from needle fainting, needle stagnation, or serious infection. In the acupuncture group, there were two cases of local bruising, but no afterpain and acid distension. In the sham acupuncture group, there was one case of local bruising after acupuncture, and three cases of residual pain or acid distension.

## 4. Discussion

In this study, the resting neuroimaging method was used to observe the brain function changes of acupuncture at Zuqiaoyin (GB44) in the treatment of migraine without aura. The ALFF method was used to study and analyze the fMRI data before and after acupuncture so as to preliminarily explore the analgesic effect center of acupuncture at GB44 in the treatment of migraine without aura. In acupuncture studies, nonacupoints are considered to have no therapeutic effect and have often been used as placebo controls in previous clinical trials and neuroimaging studies [29, 30]. Meanwhile, nonacupoints are also a proven sham acupuncture control method with the advantage of minimizing patient bias [31, 32]. Therefore, we chose the midpoint of the line between Fenglong (ST40) and Chengshan (BL57) as the nonacupoints of the sham acupuncture group. Considering the influence of patients′ expectations on brain activity and clinical efficacy [33, 34], we evaluated the reliability and expectancy of acupuncture before patients were enrolled to ensure that different expectations of acupuncture and moxibustion would not affect the outcome evaluation of patients in the two groups.

### 4.1. Comparison of Clinical Efficacy Between the Two Groups

Based on the clinical results of our study, we found that acupuncture at GB44 and nonacupoints can both relieve the clinical symptoms, while acupuncture at GB44 is superior to acupuncture at nonacupoints in reducing the frequency, days of migraine attacks, and improving quality of life. We speculated that the improvement of clinical symptoms in the sham acupuncture group might be caused by the comforting effect of acupuncture. Doctor–patient communication is an important factor in the placebo effect. Several studies [35, 36] have shown that different degrees of communication between doctors and patients would lead to different improvements of patients′ clinical symptoms and better communication can make patients′ improvement rate of various clinical symptoms higher. In this study, two acupuncturists talked with patients about migraine during the acupuncture intervention, which may explain the improvement in clinical outcomes among patients in the sham acupuncture group.

### 4.2. Comparison of Brain Activity Between the Two Groups

By comparing the brain regions of the two groups after acupuncture intervention, it was found that the acupuncture group has a higher level of brain activity in the right precentral gyrus and right precuneus. We also found that acupuncture at GB44 can enhance the brain activity of the left postcentral gyrus and left precuneus and decrease the brain activity in the right superior frontal gyrus, right insula, and right precuneus, while acupuncture at nonacupoints only decreased the brain activity in the left superior frontal gyrus and left precuneus.

The precuneus is a part of the parietal lobule, located in the medial hemisphere of the brain. It is associated with high‐level cognitive functions, such as episodic memory and self‐awareness regulation. Abnormal function of the precuneus can lead to negative emotions, such as anxiety, sadness, and worthlessness [37]. The precuneus participates in the cognition and evaluation of migraine through the adjustment of pain sensitivity. Several studies have shown that pain sensitivity was negatively correlated with functional activity in the precuneus in healthy adults [38, 39]. In addition, the precuneus is the core region of the default mode network (DMN). Previous resting state fMRI studies have shown that various pain disorders are related to abnormal connectivity patterns between DMN regions [40]. In our study, fALFF values in the precuneus were changed in both groups, and fALFF values were higher in the acupuncture group, suggesting that acupuncture analgesia can be achieved by adjusting the activity level of precuneus brain in migraine patients. This is similar to the results of previous fMRI studies [41, 42].

Previous studies have confirmed that the superior frontal gyrus plays a key role in emotional response and participatory sensation of pain, including attentional response, memory, and cognitive response related to pain [43, 44]. The prefrontal cortex (PFC) consists of superior frontal gyrus, middle frontal gyrus, and inferior frontal gyrus, which refers to the entire frontal cortex except the primary and secondary motor cortex. It is the regulatory region associated with opioid analgesia and other forms of analgesia and may reduce the perception of pain signals through cognitive regulation [45–47]. Previous studies on pain‐induced stimulation [48, 49] found that migraine patients had more activation in the PFC. Thus, enhanced pain sensations may affect brain sensitivity, particularly in the superior frontal gyrus. Our clinical results suggest that acupuncture may relieve headache to varying degrees, which may be one of the reasons for the change of brain activity in superior frontal gyrus in migraine patients after acupuncture.

The posterior central gyrus is the primary somatosensory cortex, one of the components of the pain matrix, mainly involved in pain discrimination and processing [50]. Previous studies have found that the functional abnormalities of somatosensory centers in migraine patients were related to the changes in cortical thickness and gray matter volume [51, 52]. Meanwhile, migraine patients also showed increased glucose metabolism in the posterior central gyrus [53]. These studies have demonstrated that migraine is closely related to structural and functional changes in the posterior central gyrus. As the primary motor cortex, the anterior central gyrus is considered to be an important node in processing pain signals [54]. Some studies have found a close link between the anterior central gyrus and posterior central gyrus, suggesting that some sensory signals can be transmitted from primary somatosensory cortex to primary motor cortex [55]. In our study, the fALFF value of the posterior central gyrus was changed after acupuncture at GB44 and significant differences in fALFF of the anterior central gyrus were found between the two groups after comparison of brain regions, indicating that acupuncture at GB44 can regulate the activity level of the primary somatosensory cortex and cause corresponding activity changes of the primary motor cortex indirectly.

The insula is a cortical center buried in the lateral sulci and is involved in many important brain functions, including goal‐directed cognition, conscious perception, and autonomic regulation. The insula may be the “active center” of migraine onset [56]. Studies on pain have confirmed the existence of right‐side somatosensory dominance in insula activation. In a PET‐CT study [57] on spontaneous migraine, right‐side dominant activation in insula exists and local remodeling in insula leads to changes in the degree of spontaneous neuronal activity after repeated attacks [58]. Similar to the results of previous studies, all patients in this study were right‐handed and acupuncture at GB44 could cause changes in brain activity of insula.

### 4.3. Limitation

In our study, some limiting factors should be considered. First, to further explore the relationship between neuroimaging changes and clinical outcomes, we performed Pearson correlation analyses between fALFF changes within the significant clusters and changes in clinical scores. However, no significant correlations were observed (all *p* > 0.05), which may be attributable to the relatively small sample size and insufficient statistical power. In the next study, emphasis should be placed on the correlation analysis and the influence of acupuncture therapy on pain‐related brain functional networks such as the DMN to further explore its central mechanism. Second, due to time limitation, the follow‐up period was not set up, which was not conducive to testing the long‐term clinical effect and neuroimaging changes of acupuncture at GB44 in the treatment of migraine without aura. Third, we recruited 20 migraine patients without aura and the results in our study need further verification in a large sample.

## 5. Conclusion

In conclusion, acupuncture at GB44 can significantly relieve the clinical symptoms and improve the quality of life of patients with migraine without aura. More importantly, acupuncture GB44 can cause changes of brain activity in postcentral gyrus, precuneus, superior frontal gyrus, and insula, which may be one of the central mechanisms of acupuncture in the treatment of migraine without aura.

## Funding

The study was funded by the Clinical Research Innovation Plan of Shanghai General Hospital (IIT2025‐168) and the Shanghai Municipal Health Commission Traditional Chinese Medicine Research Project (2024QN043).

## Disclosure

The study sponsor had no role in the design and conduct of the study; collection, management, analysis, and interpretation of the data; preparation, review, or approval of the manuscript; and decision to submit the manuscript for publication.

## Conflicts of Interest

The authors declare no conflicts of interest.

## Data Availability

The data that support the findings of this study are available from the corresponding author upon reasonable request.
